# Upregulation of centrosomal protein 55 is associated with unfavorable prognosis and tumor invasion in epithelial ovarian carcinoma

**DOI:** 10.1007/s13277-015-4419-6

**Published:** 2015-11-28

**Authors:** Weijing Zhang, Chunhao Niu, Weiling He, Teng Hou, Xiaoying Sun, Liqun Xu, Yanna Zhang

**Affiliations:** 1 State Key Laboratory of Oncology in South China, Collaborative Innovation Center for Cancer Medicine, Department of Gynecologic Oncology, Cancer Center, Sun Yat-Sen University, No. 651, Dongfeng Road East, Guangzhou, 510060 China; 2 Department of Gastrointestinopancreatic Surgery, The First Affiliated Hospital of Sun Yat-sen University, Zhongshan Second Road 58, Guangzhou, 510080 China; 3 Department of Urology, Wuhan Union Hospital of Huazhong University of Science and Technology, No.1277, Han Kou Jie Fang Road, Wuhan, 430022 China; 4 Department of Gynecology, Women and Children Hospital of Guangdong Province, No.13, Guang Yuan Road, Guangzhou, 510060 China

**Keywords:** CEP55, Ovarian cancer, Epithelial–mesenchymal transition, Prognosis, Biomarker

## Abstract

Centrosomal protein 55 (CEP55) is a cell cycle regulator implicated in development of certain cancers. However, characteristics of CEP55 expression and its clinical/prognostic significance are unclear in human epithelial ovarian carcinoma (EOC). Therefore, we investigated the expression and clinicopathological significance of CEP55 in patients with EOC and its role in regulating invasion and metastasis of ovarian cell lines. CEP55 mRNA and protein expression levels were detected by quantitative real-time PCR (qRT-PCR), Western blotting, and immunohistochemistry (IHC). Potential associations of CEP55 expression scores with clinical parameters and patient survival were evaluated. CEP55 function was investigated further using RNA interference, wound healing assay, transwell assay, immunofluorescence analysis, qRT-PCR, and Western blotting. CEP55 was significantly upregulated in ovarian cancer cell lines and lesions compared with normal cells and adjacent noncancerous ovarian tissues. In the 213 EOC samples, CEP55 protein levels were positively correlated with clinical stage (*P* < 0.001), lymph node metastasis (*P* < 0.001), intraperitoneal metastasis (*P* < 0.001), tumor recurrence (*P* < 0.001), differentiation grade (*P* < 0.001), residual tumor size (*P* < 0.001), ascites see tumor cells (*P* = 0.020), and serum CA153 level (*P* < 0.001). Moreover, patients with aberrant CEP55 protein expression showed tendencies to receive neoadjuvant chemotherapy (*P* < 0.001) and cytoreductive surgery (*P* = 0.020). By contrast, no significant correlation was detected between the protein levels and patient age, histological type, or serum CA125, CA199, CA724, NSE, CEA, and β-HCG levels. Patients with high CEP55 protein expression had shorter overall survival and disease-free survival compared with those with low CEP55 expression. Multivariate analysis implicated CEP55 as an independent prognostic indicator for EOC patients. Additionally, downregulation of CEP55 in ovarian cancer cells remarkably inhibited cellular motility and invasion. Aberrant CEP55 expression may predict unfavorable clinical outcomes in EOC patients and play an important role in regulating invasion in ovarian cancer cells. Thus, CEP55 may serve as a prognostic marker and therapeutic target for EOC.

## Introduction

Ovarian cancer is one of the most lethal gynecologic malignancies and is the leading cause of gynecological cancer death [1]. There were 204,000 new cases and 125,000 deaths estimated worldwide in 2011 [2]. Although advances in surgery and new chemotherapy regimens have resulted in downward trends of the incidence and mortality of ovarian cancer over the last few decades, it is still associated with the highest mortality rate among all gynecological malignancies worldwide [3]. One reason for the lethality of ovarian cancer is that the majority of women are undiagnosed until advanced International Federation of Gynecology and Obstetrics (FIGO) stages (III or IV) where the cancer has spread beyond the pelvis, which leads to an unfavorable prognosis [4]. Several traditional clinical variables, including surgical stage, volume of residual tumor after primary surgery, and histologic grade play important roles in the FIGO staging system and patient prognosis. Moreover, biomarkers such as CA125, CA199, and CA153 have been used for predicting metastasis and prognosis in the clinic [5]. Many novel genes, such as *AGR2*, *Netrin-1* and *STIP1*, have been reported to be potentially useful metastatic and prognostic markers in ovarian cancer [6–8]. However, they are not sufficiently reliable for predicting tumor metastasis, clinical outcomes or for optimizing and individualizing the treatment. Thus, an urgent need remains for additional research to identify novel biomarkers for developing targeted therapy, detection of metastasis, and predicting the survival and relapse rates for ovarian cancer patients.

Centrosomal protein 55 (CEP55), also designated as C10orf3, FLJ10540, or URCC6, is a centrosome- and midbody-associated protein of ~55 kDa in size and has been mapped to the 10q23 chromosomal region [9]. The *CEP55* gene encodes the 464 amino acid protein containing a domain known as AAA (ATPases associated with a variety of cellular activities). CEP55 was found to play a role in centrosome-dependent cellular functions, such as centrosome duplication and/or cell cycle progression, or in the regulation of cytokinesis [10–13]. Recently, increased expression of CEP55 was reported in several human tumors, and it may be associated with the onset of oncogenesis, invasion, and mitosis. A study indicated that CEP55 forms a complex with PI3K, enhancing PI3K activity, and consequently, the AKT survival pathway, suggesting that CEP55 is a novel oncogene that may play an important role in hepatocarcinogenesis [14]. A higher level of CEP55 has been associated with poor prognosis in ER+ breast cancer patients [15]. It is reported that the early upregulation of FOXM1 during head and neck cancer progression, rendering it as an attractive diagnostic biomarker for early cancer detection and its candidate mechanistic targets, CEP55 and HELLS, as indicators of malignant conversion and progression [16]. In addition, expression of CEP55 was correlated with aggressiveness of oral cavity squamous cell carcinoma by stimulating cell migration and invasion through increased FOXM1 and MMP-2 activity [17]. CEP55 was reported to be overexpressed in lung cancer tissues and associated with cell migration and invasion, as well as participate in the VEGF-A/PI3K/AKT pathway [18, 19]. Furthermore, some studies have identified the overexpression of CEP55 in colorectal carcinoma, prostate cancer, nasopharyngeal carcinoma, and gastric cancer [20–26]. However, characteristics of CEP55 expression and its clinical/prognostic significance in human ovarian cancer remain unknown.

In the current study, we aimed to explore the expression of CEP55 in ovarian cell lines and human ovarian tissues. Moreover, we investigated the association between the expression of CEP55 protein and clinical manifestations and survival outcomes of a cohort of 213 patients with ovarian cancer. We further investigated the function of CEP55 by using RNA interference (RNAi), wound healing assay, Transwell assay, immunofluorescence analysis, quantitative real-time PCR (qRT-PCR), and Western blot analysis, .

## Methods

### Samples and patients

This study was approved by the Sun Yat-sen University Cancer Center Ethic Review Committee, and each patient signed an informed consent prior to the use of the clinical materials for research purposes. All specimens were handled according to the ethical and legal standards. For qRT-PCR and Western blot analysis, fresh ovarian cancer and matched distant noncancerous ovarian tissues were derived from 12 patients who had undergone surgery at the Sun Yat-sen University Cancer Center between March 2015 and May 2015. For Western blot analysis of different stages of ovarian cancer, we collected normal ovarian tissue, benign ovarian cancer tissue, and borderline ovarian cancer and ovarian cancer tissues from patients at different clinical stages from seven patients who had undergone surgery at the Sun Yat-sen University Cancer Center between January 2015 and June 2015. In addition, immunohistochemical (IHC) analysis was conducted on a total of 213 paraffin-embedded ovarian cancer samples, which were histopathologically and clinically diagnosed at the Sun Yat-sen University Cancer Center between 2002 and 2010. Clinical and clinicopathological classification and staging were determined by two experienced gynecological oncologists according to FIGO (2009). The follow-up time for the primary ovarian cancer cohort ranged from 5.1 to 176.1 months with a median of 73.82 months. The clinicopathological data of all the patients were summarized in Table 1.

**Table 1:** Clinicopathological characteristics and tumor expression of *CEP55* in patients with EOC

Characteristic	Number of cases (%) (%)
Age (years)	
<53	111 (52.1)
≥53	102 (47.9)
FIGO stage	
I	59 (27.7)
II	36 (16.9)
III	100 (46.9)
IV	18 (8.5)
Histological type	
Serous adenocarcinoma	154 (72.3)
Mucoid adenocarcinoma	44 (20.7)
Endometrial adenocarcinoma	9 (4.2)
Clear cell carcinoma	6 (2.8)
Lymph node metastasis	
Absent	173 (81.2)
Present	40 (18.8)
Intraperitoneal metastasis	
No	110 (51.6)
Yes	103 (48.4)
Expression of C14ORF166	
Low or none	114 (53.5)
High	99 (46.5)
Tumor recurrence	
No	121 (56.8)
Yes	92 (43.2)
Vital status (at last follow-up)	
Alive	111 (52.1)
Dead	102 (47.9)
Differentiation grade	
G1/G2	128 (60. 1)
G3	85 (39.9)
Residual tumor size (cm)	
≤1	150 (70.4)
>1	63 (29.6)
Neoadjuvant chemotherapy	
No	158 (74.2)
Yes	55 (25.8)
Postoperative chemotherapy	
No	22 (10.3)
Yes	191 (89.7)
HIPEC	
No	142 (66.7)
Yes	71 (33.3)
Ascites see tumor cells (+)	
No	148 (69.5)
Yes	65 (30.5)
Cytoreductive surgery	
No	71 (33.3)
Yes	142 (66.7)
CA125 (U/ml)	
≤35	14 (6.6)
>35	197 (93.4)
CA199 (U/ml)	
≤35	55 (26.1)
>35	156 (73.9)
CA153 (U/ml)	
≤25	82 (40.4)
>25	121 (59.6)
NSE (U/ml)	
≤15.2	46 (31.5)
>15.2	100 (68.5)
CEA (U/ml)	
≤5.0	67 (83.8)
>5.0	13 (16.2)
β-HCG (U/ml)	
≤3.0	65 (71.4)
> 3.0	26 (28.6)
CA724 (U/ml)	
≤5.3	5 (38.5)
>5.3	8 (61.5)

*HIPEC* hyperthermic intraperitoneal chemotherapy, *FIGO* International Federation of Gynecology and Obstetrics

### Cell lines

The ovarian cancer cell lines used in the current study were obtained from American Type Culture Collection (ATCC, Manassas, VA). TOV-112D, COV434, OV-90, COV644, COV504, COV362, A2780, TOV-21G, SKOV3, OVCAR4, and EFO-27 were cultured in Dulbecco’s modified Eagle’s medium (DMEM) (Gibco, Grand Island, NY, USA) supplemented with 10 % fetal bovine serum (FBS, HyClone, Logan, UT, USA) and 1 % antibiotics (100 U/ml penicillin and 100 ug/ml streptomycin). The normal ovarian cell line HOSEpiC was maintained in DMEM.

### qRT-PCR analysis

qRT-PCR was performed to detect the expression levels of CEP55 in ovarian cell lines, human epithelial ovarian cancer, and matched adjacent normal tissues. Total RNA samples from cultured cells and fresh tissues were isolated using TRIzol reagent (Invitrogen, Carlsbad, CA, USA) according to the manufacturer’s instructions. These RNA samples were then pretreated with RNase-free DNase, and 2 μg of RNA sample was used for cDNA synthesis using random hexamers. The primers were designed using Primer Express v2.0 (Applied Biosystems, USA). CEP55 primers used were 5′-GCCACTGCTGATTTTTCTCC-3′, and 5′-ACTGTGGCTCCAAACTGCTT-3′.

For the *glyceraldehyde-3-phosphate dehydrogenase* (GAPDH) gene, which was used as an internal control, the sense primer 5′-AATGAAGGGGTCATTGATGG-3′ and the antisense primer 5′-AAGGTGAAGGTCGGAGTCAA-3′ were used. For PCR-mediated amplification of *CEP55* cDNA, an initial amplification step using *CEP55*-specific primers was conducted with a denaturation step at 95 °C for 10 min, followed by 30 denaturation cycles at 95 °C for 60 s, primer annealing at 55 °C for 30 s, and a primer extension phase at 72 °C for 30 s. Upon the completion of the cycling steps, a final extension at 95 °C was carried out before the reaction mixture was held at 4 °C. qRT-PCR was then performed to evaluate the fold increase of *CEP55* mRNA in each of the primary ovarian cancer tissues relative to the matched adjacent normal tissues (ANT) and ovarian cancer cell lines relative to that in the normal ovarian cell line. We used a double-stranded DNA-specific SYBR Premix Ex Taq II kit (Takara Biotechnology, USA) on a Bio-Rad sequence evaluation system according to the manufacturer’s instructions. The expression data were normalized to the geometric mean of the level for GAPDH to control for variability in expression levels in all experiments. All assays were examined in triplicate.

### Western blotting

Total protein was prepared using the cell total protein extraction kits according to the manufacturer’s instruction (Millipore, Billerica, MA). Equal concentrations of each protein sample (20 μg) were separated on 6 % SDS polyacrylamide gels and transferred to polyvinylidene fluoride (PVDF) membranes (Immobilon P, Millipore, Bedford, MA, USA). The membranes were blocked with 5 % nonfat milk in Tris-buffered saline containing 0.1 % Tween 20 (TBST) for 1 h at room temperature, and incubated a primary monoclonal antibody to CEP55 (1:1000, Abcam, USA, ab170414) overnight at 4 °C. After washing with TBST, the membranes were incubated with horseradish peroxidase-conjugated goat anti-rabbit IgG (Santa Cruz Biotechnology, Dallas, TX, USA, SC-2004). The ECL prime Western blotting detection reagent (Amersham Pharmacia Biotech, Piscataway, NJ) was used to detect CEP55 expression according to the manufacturer’s instructions. GADPH (Santa Cruz Biotechnology) was used to confirm equal loading of the samples.

### IHC analysis

IHC analysis was carried out to investigate alterations in protein expression in the 213 human ovarian cancer tissues. The procedures were performed with classical protocols. In brief, paraffin-embedded specimens were cut into 4-μm sections and baked at 65 °C for 30 min. The sections were deparaffinized with xylene and rehydrated. Sections were submerged into EDTA antigenic retrieval buffer, microwaved for antigenic retrieval, and then treated with 3 % hydrogen peroxide in methanol to quench endogenous peroxidase activity, followed by incubation with 1 % bovine serum albumin (BSA) to block any nonspecific binding. Sections were then incubated with a rabbit monoclonal antibody against CEP55 (Abcam, ab170414, 1:250) overnight at 4 °C. After washing, the tissue sections were then incubated with a biotinylated anti-rabbit secondary antibody (Abcam), followed by further incubation with a streptavidin-horseradish peroxidase complex (Abcam). The tissue sections were immersed in 3-amino-9-ethyl carbazole and counterstained with 10 % Mayer’s hematoxylin, dehydrated and mounted in Crystal Mount.

The degree of immunostaining of formalin-fixed, paraffin-embedded sections was blind reviewed and evaluated by two independent pathologists. Scores given by the two independent observers were averaged, which were based on both the proportion of positively stained tumor cells and the intensity of staining. The proportion of tumor cells was scored as follows: 1 (<10 % positive tumor cells), 2 (10–50 % positive tumor cells), 3 (50–75 % positive tumor cells), and 4 (>75 % positive tumor cells). Cells were scored for intensity of staining on a scale of 0 (no staining), 1 (weak staining = light yellow), 2 (moderate staining = yellow brown), and 3 (strong staining = brown). The staining index was calculated as the product of the proportion of positive cells × staining intensity score (range from 0 to 12). Cutoff values for CEP55 were chosen on the basis of a measure of heterogeneity using the log-rank test with respect to overall survival (OS). An optimal cutoff value was identified as follows: staining index score of ≥6 was used to define tumors with high CEP55 expression, and a score of ≤4 indicated low CEP55 expression.

### RNAi and transfection

To further identify the role of CEP55 in tumor invasion, two human small interfering RNAs (siRNAs) were synthesized and purified by Ribobio Inc (Guangzhou, Guangdong, China) for depletion of CEP55. The siRNA sequences used were as follows: siRNA#1: 5′-GAAGCCUAGUAACUCCAAAdTdT-3′, siRNA#2: 5′-GGAAGAUGAUAGGCAUAAAdTdT-3′, and siRNA#3: 5′-GGAAACAGCUGCUCAUUCAdTdT-3′. Transfection of siRNAs was carried out using Lipofectamine 2000 reagent (Invitrogen) according to the instructions of the manufacturer.

### Cell invasion assay

In the cell invasion assay, we used Transwell chambers (Costar, Cambridge, MA, USA) with 8-μm pore polycarbonate filters that were coated with Matrigel^™^ (BD Biosciences, Franklin Lakes, NJ, USA). Cells cultured in the absence or presence of siRNA for 24 h were seeded into the upper chambers in serum-free medium at a density of 2.5 × 10^4^ per well, and 500 μl of NIH3T3-conditioned medium was placed in the lower chamber as a chemoattractant. After 48 h at 37 °C in 5 % CO_2_, the cells that passed through the filter into the lower chamber were fixed with 3.7 % paraformaldehyde and stained with 0.1 % crystal violet solution. Cells on the upper surface of the filter were removed with cotton buds. Invaded cells on the underside of the filter were photographed and counted by phase contrast microscopy (×200 magnification). The experiments were performed in triplicate.

### Wound-healing assay

For wound-healing assay, cells were seeded in individual wells of a six-well culture plate. Untransfected cells and cells exposed to CEP55 siRNA or negative control siRNA for 5 h were cultured in a quiescent medium for 24 h. Thereafter, a sterile 10-μl pipette tip was used to longitudinally scratch a constant-diameter stripe in the confluent monolayer. The medium and cell debris were removed by aspiration and replaced with 2 ml of fresh serum-free medium. Microscopic images were taken at 0, 24, and 48 h immediately after wounding (corresponding to 24, 48, and 72 h posttransfection) by phase contrast microscopy. For statistical analysis, three randomly selected points along each wound were marked, and the horizontal distance between the migrating cells and the initial wound was measured at 24 and 48 h. Values were means ± standard deviation (SD) from at least three independent experiments. Differences between siRNA-treated and blank control data were determined by a Student’s *t* test, where *P* < 0.05 was considered significant.

### Immunofluorescence analysis

After a 5-h exposure to CEP55 siRNA, negative control siRNA, or no treatment, SKOV3 and TOV-21G cells were cultured overnight on sterile glass coverslips in a six-well plate for 24 h. The cells were washed in PBS, fixed in 3.7 % paraformaldehyde, permeabilized in 0.1 % Triton-X100, and incubated in blocking solution (1 % BSA). The cells were then incubated with primary antibodies against E-cadherin, N-cadherin, β-catenin, or fibronectin (BD Transduction Laboratories, Lexington, UK) and then incubated with rhodamine-conjugated goat antibodies against rabbit or mouse IgG (Jackson ImmunoResearch Laboratories, USA). The coverslips were counterstained with DAPI (Sigma-Aldrich, St Louis, MO) and imaged with a confocal laser-scanning microscope (Olympus FV1000, Japan). Data were processed with Adobe Photoshop 7.0.

### Statistical analysis

All statistical analyses were carried out using the SPSS 16.0 statistical software packages. The associations of clinicopathological characteristics with the expression status of CEP55 were assessed using the chi-square test and Fisher’s exact test. Bivariate correlations between studied variables were calculated by Spearman’s rank correlation coefficients. Survival curves were plotted by the Kaplan–Meier method, and differences were analyzed using the log-rank test. Multivariate analysis was performed using the Cox proportional hazards regression model on all significant characteristics determined using univariate analysis. All *P* values were two-sided. A *P* value of <0.05 was considered to be statistically significant in all cases.

## Results

### CEP55 is upregulated in ovarian cancer

**Fig. 1:**
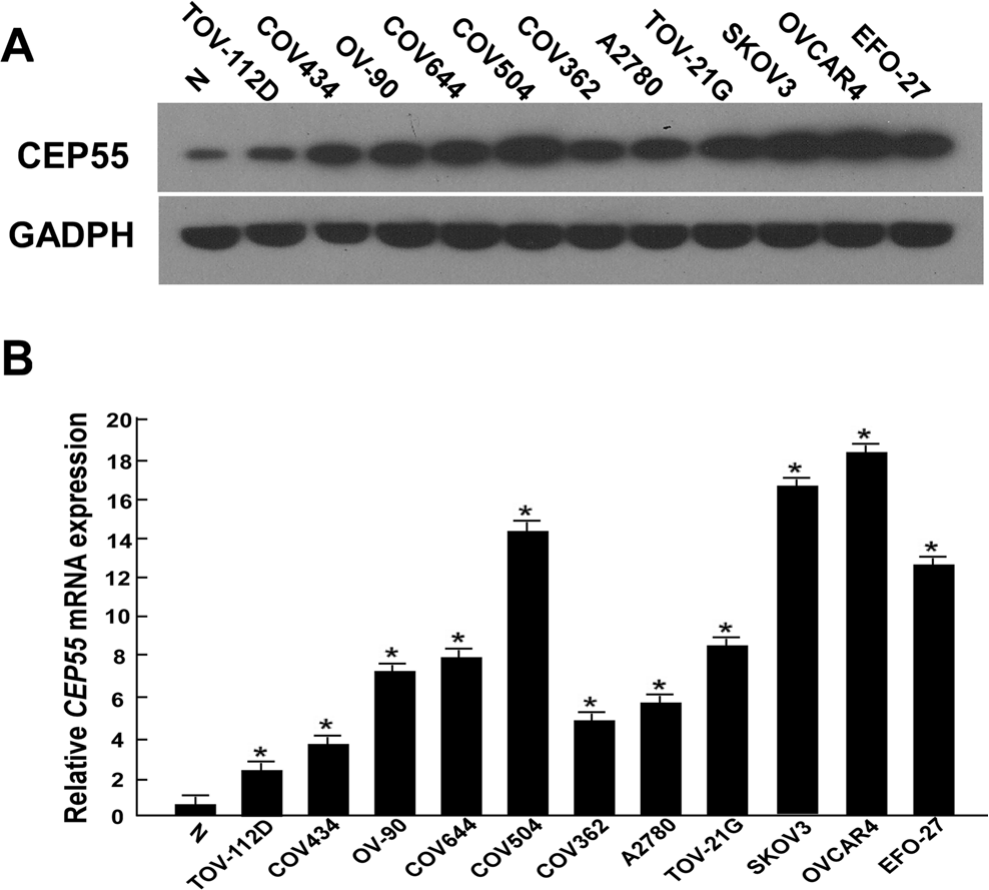
Overexpression of CEP55 mRNA and protein in ovarian cancer cell lines. **a**, **b** Expression of CEP55 mRNA and protein in ovarian cancer cell lines (TOV-112D, COV434, OV-90, COV644, COV504, COV362, A2780 and TOV-21G, SKOV3, OVCAR4, EFO-27) and the normal ovarian cell line HOSEpiC (N) were examined by Western blotting (**a**) and qRT-PCR (**b**). Expression levels were normalized against GAPDH. Results are shown as the fold increase in *CEP55* mRNA expression relative to that of normal ovarian cells. Error bars represent the SD of the mean calculated from three parallel experiments (*P < 0.05)

**Fig. 2:**
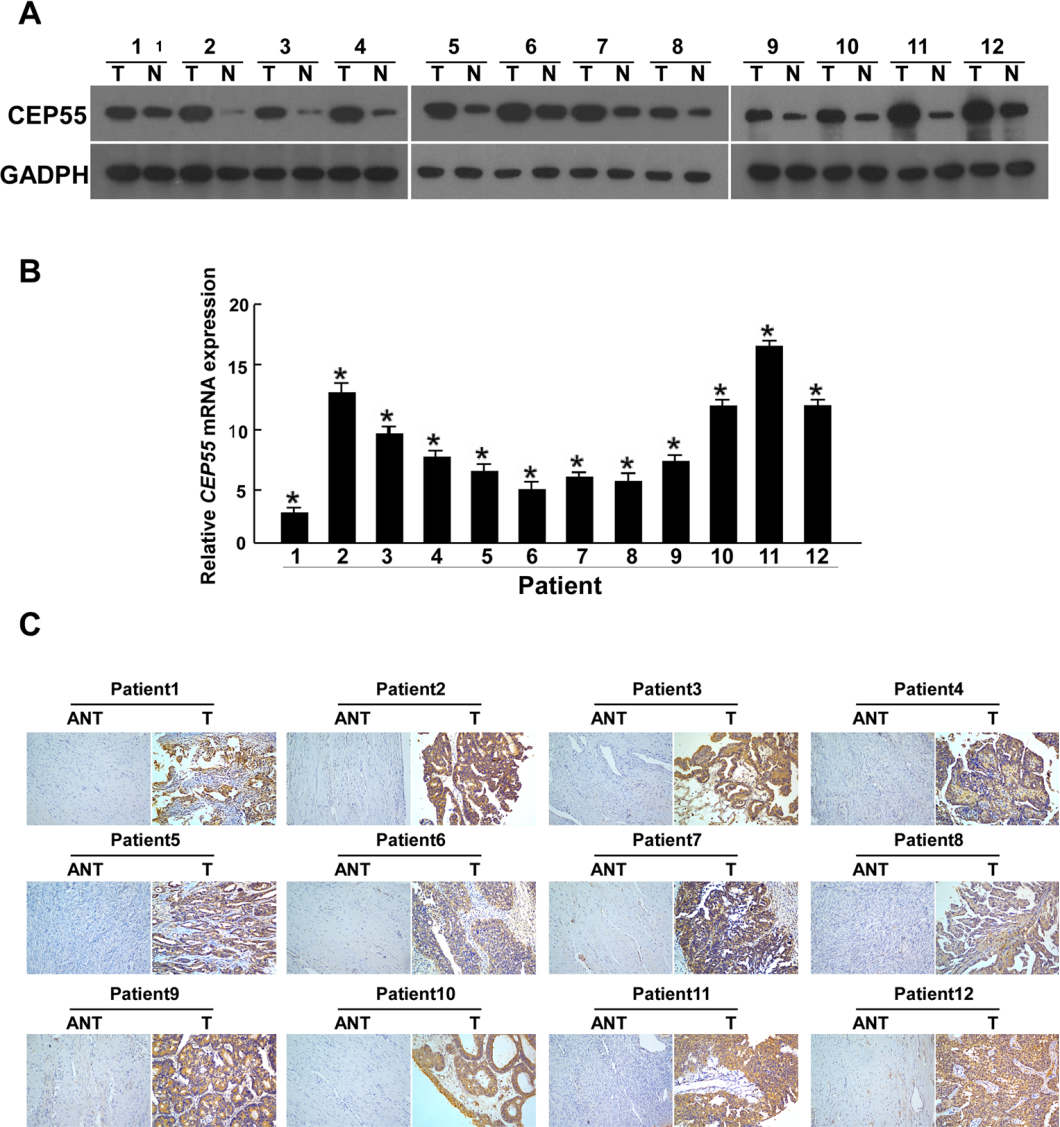
Overexpression of CEP55 mRNA and protein in EOC tissues. **a** Representative images of Western blotting analyses of CEP55 protein expression in 12 matched pairs of ovarian cancer (T) and adjacent noncancerous tissues. The expression level was normalized by GADPH expression. **b** Average T/N ratios of *CEP55* mRNA expression in paired ovarian cancer (T) and adjacent noncancerous tissues (N) were quantified by qRT-PCR and normalized against *GAPDH*. Results are shown as the fold increase in *CEP55* mRNA expression relative to that of adjacent noncancerous tissues (N). Error bars represent the SD of the mean calculated from three parallel experiments. **c** Immunohistochemical assay of CEP55 protein expression in 12 pairs of matched ovarian cancer tissues (*P < 0.05)

**Fig. 3:**
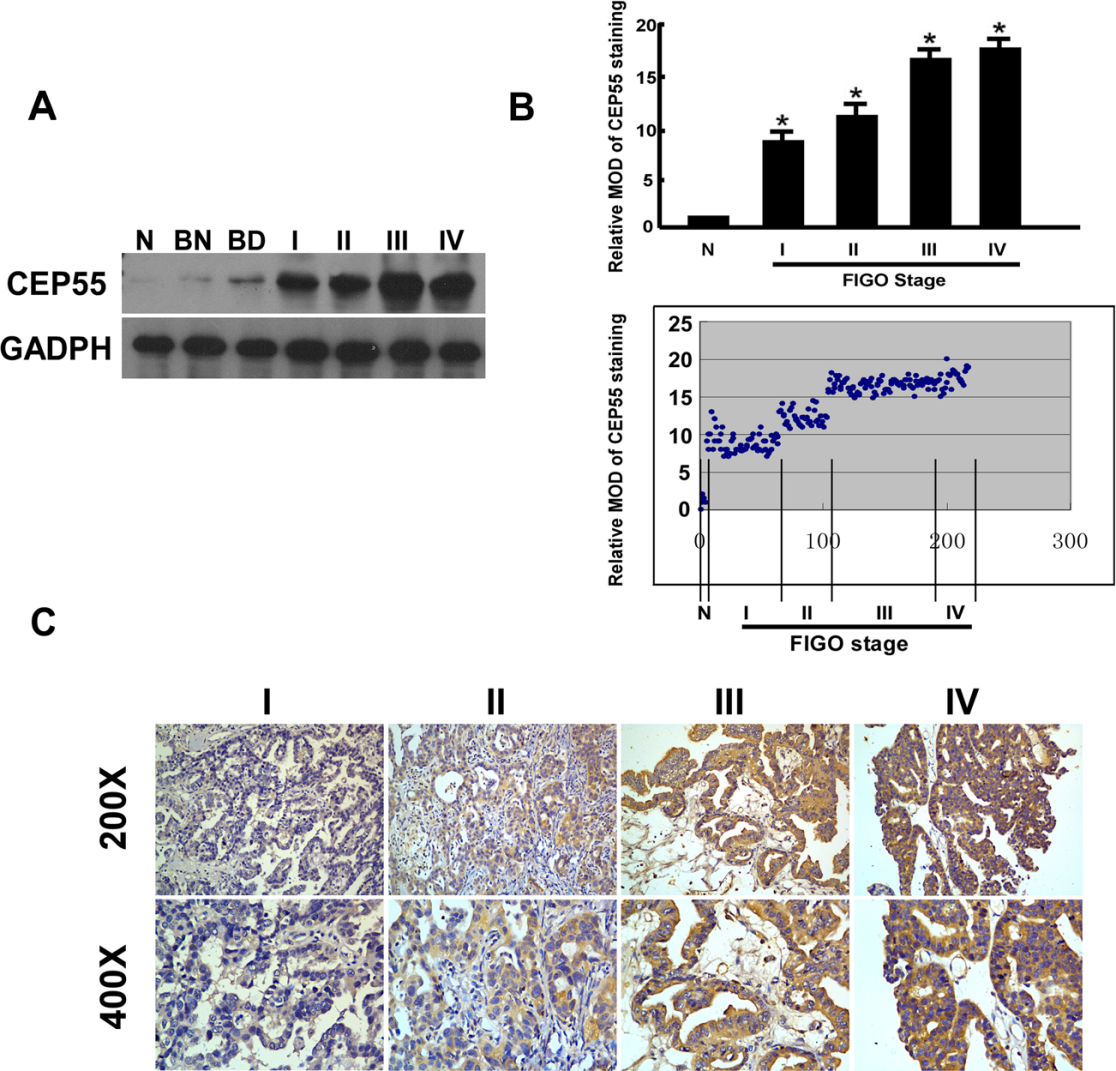
The expression of CEP55 in epithelial ovarian cancer tissues from patients at different clinical stages. **a** Representative images of Western blotting analyses of CEP55 protein expression in normal ovarian tissue, benign ovarian cancer tissue, and borderline ovarian cancer and ovarian cancer tissues from patients at different clinical stages. **b** Statistical analyses of the average MOD of CEP55 staining in normal ovarian tissue and ovarian cancer specimens at different clinical stages. Values represent the mean ± SD from three independent experiments. Results are shown as the fold increase in MOD of CEP55 staining relative to that of normal ovarian tissue (*P < 0.05)*.* **c** Representative images from immunohistochemical analyses of CEP55 expression in ovarian cancer tissues at different clinical stages

Western blot and qRT-PCR analyses revealed high levels of CEP55 expression in all 11 ovarian cell lines tested compared with the immortalized normal ovarian epithelial cell line HOSEpiC (Fig. 1a, b). In order to determine whether the CEP55 upregulation found in ovarian cancer cell lines was related to clinical biochemical indicators, we did Western blotting analysis on 12 paired epithelial ovarian carcinoma tissues and noncancerous tissues adjacent to ovarian tumors. As shown in Fig. 2a, b, CEP55 protein expression was higher in all 12 epithelial ovarian carcinoma samples, displaying more than a threefold increase compared with that in the adjacent noncancer tissue samples. These findings above are consistent with the results obtained in our immunohistochemical analysis (Fig. 2c). Moreover, CEP55 was shown to be predominantly located in the cytoplasm of tumor cells (Figs. 2c and 3c). As shown in Fig. 3a, Western blotting results indicated that CEP55 protein expression was low in normal ovarian tissue, benign ovarian cancer tissue, and borderline ovarian cancer tissue, while it was high in ovarian carcinoma tissues from patients at different clinical stages. Interestingly, Western blot analysis showed that CEP55 protein expression levels were positively correlated with clinical stages of ovarian carcinoma patients.

### CEP55 overexpression is associated with clinical features of ovarian cancer

**Fig. 4:**
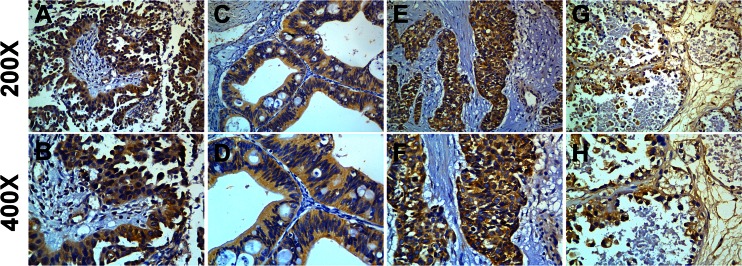
Representative images from immunohistochemical analyses of CEP55 expression in different histotypes of EOC specimens. **a**, **b** High expression in serous histotypes. **c**, **d** High expression in mucinous histotypes. **e**, **f** High expression in endometrioid histotypes. **g**, **h** High expression in clear cell histotypes

To further determine whether CEP55 protein overexpression is associated with clinicopathological characteristics of epithelial ovarian cancer, 213 paraffin-embedded, archived ovarian cancer tissue samples were examined by immunohistochemical analysis. The samples included 59 cases at stage I, 36 cases at stage II, 100 cases at stage III, and 18 cases at stage IV. In the cohort, CEP55 expression in 213 enrolled patient samples was determined as strong in 99 cases (46.5 %) and weakly positive or negative in 114 cases (53.5 %) (Table 1). As shown in Fig. 4, the immunoreactivity of CEP55 was detected at variable levels, and specific CEP55 staining was mostly found in the cytoplasm of carcinoma cells. Additionally, the CEP55 protein expression was generally weak in early stage ovarian cancer (FIGO stages I and II), while it was strong in later stage ovarian cancer (FIGO stages III and IV) tissues (Fig. 3c). Quantitative analysis indicated that the mean optical density (MOD) values of CEP55 staining in clinical stage I–IV primary tumors were statistically significantly higher than those in the normal control ovarian tissues. In addition, the MOD values of CEP55 staining significantly increased with progression from stage I to IV (*P* < 0.001, Fig. 3b). Taken together, these observations suggested that aberrant CEP55 expression was associated with the clinical development of primary ovarian tumors.

Statistical analyses were employed to detect the correlation between CEP55 expression and the clinicopathological characteristics of ovarian cancer patients (Table 1). As summarized in Table 2, no significant correlations were detected between the expression level of CEP55 protein and patient age, histological type, and serum CA125, CA199, NSE, CEA, β-HCG, or CA724 expression levels in patients with ovarian cancer. However, CEP55 expression was markedly associated with clinical stage (*P* < 0.001), lymph node metastasis (*P* < 0.001), intraperitoneal metastasis (*P* < 0.001), tumor recurrence (*P* < 0.001), differentiation grade (*P* < 0.001), residual tumor size (*P* < 0.001), ascites see tumor cells (*P* = 0.020), and serum CA153 level (*P* < 0.001) in patients with ovarian cancer. Moreover, patients with aberrant CEP55 protein expression showed tendencies to receive neoadjuvant chemotherapy (*P* < 0.001) and cytoreductive surgery (*P* = 0.020). These data were further confirmed by association coefficient analyses assessing the correlation between CEP55 expression and clinicopathological features. As shown in Table 3, correlations between CEP55 expression and clinical stage, lymph node metastasis, intraperitoneal metastasis, tumor recurrence, differentiation grade, residual tumor size, serum CA153 level, and ascites see tumor cells were 0.550 (*P* < 0.001), 0.328 (*P* < 0.001), 0.414 (*P* < 0.001), 0.607 (*P* < 0.001), 0.251 (*P* < 0.001), 0.235 (*P* < 0.001), 0.289 (*P* < 0.001), and 0.157 (*P* = 0.020), respectively.

**Table 2:** Correlation between CEP55 expression and clinicopathologic features of EOC

Characteristics		Total	CEP55		Chi-squared test *P* value	Fisher’s exact test *P* value
			No or weak expression	Moderate or strong expression		
Age (years)	<53	111	59 (27.7)	52 (24.4)	0.911	1.000
	≥53	102	55 (25.8)	47 (22.1)		
FIGO stage	I	59	56 (26.3)	3 (1.4)	<0.001	–
	II	36	29 (13.6)	7 (3.3)		
	III	100	27 (12.7)	73 (34.3)		
	IV	18	2 (0.9)	16 (7.5)		
Lymph node metastasis	Absent	173	107 (50.2)	66 (31.0)	<0.001	<0.001
	Present	40	7 (3.3)	33 (15.5)		
Histological type	Serous cell carcinoma	154	82 (38.5)	72 (33.8)	0.143	–
	Mucous cell carcinoma	44	20 (9.4)	24 (11.3)		
	Endometrial carcinoma	9	7 (3.3)	2 (0.9)		
	Clear cell carcinoma	6	5 (2.3)	1 (0.5)		
Intraperitoneal metastasis	No	110	83 (39.0)	27 (12.7)	<0.001	<0.001
	Yes	103	31 (14.5)	72 (33.8)		
Tumor recurrence	No	121	105 (49.3)	16 (7.5)	<0.001	<0.001
	Yes	92	9 (4.2)	83 (39.0)		
Vital status (at last follow-up)	No	111	95 (44.6)	16 (7.5)	<0.001	<0.001
	Yes	102	19 (8.9)	83 (39.0)		
Differentiation grade	G1/G2	128	82 (38.5)	46 (21.6)	<0.001	<0.001
	G3	85	32 (15.0)	53 (24.9)		
Residual tumor	≤1	150	92 (43.2)	58 (27.2)	<0.001	0.001
Size (cm)	>1	63	22 (10.3)	41 (19.3)		
Neoadjuvant chemotherapy	No	158	96 (45.1)	62 (29.1)	<0.001	<0.001
	Yes	55	18 (8.4)	37 (17.4)		
Postoperative chemotherapy	No	22	11 (5.2)	11 (5.2)	0.727	0.823
	Yes	191	103 (48.3)	88 (41.3)		
HIPEC	No	142	78 (36.6)	64 (30.1)	0.560	0.564
	Yes	71	36 (16.9)	35 (16.4)		
Ascites see tumor cells (+)	No	148	87 (40.8)	61 (28.6)	0.020	0.025
	Yes	65	27 (12.7)	38 (17.8)		
Cytoreductive surgery	No	71	46 (21.6)	24 (11.3)	0.020	0.021
	Yes	142	68 (31.9)	74 (34.7)		
CA125 (U/ml)	≤35	14	10 (4.7)	4 (1.9)	0.165	0.267
	>35	197	103 (48.8)	94 (44.6)		
CA199 (U/ml)	≤35	156	83 (39.3)	73 (34.6)	0.686	0.754
	>35	55	31 (14.7)	24 (11.4)		
CA153 (U/ml)	≤25	82	59 (29.1)	23 (11.3)	<0.001	<0.001
	>25	121	50 (24.6)	71 (35.0)		
NSE (U/ml)	≤15.2	46	27 (18.5)	19 (13.0)	0.387	0.475
	>15.2	100	51 (34.9)	49 (33.6)		
CEA (U/ml)	≤5.0	67	41 (51.3)	26 (32.5)	0.621	0.759
	>5.0	13	7 (8.7)	6 (7.5)		
β-HCG (U/ml)	≤3.0	65	35 (38.5)	30 (33.0)	0.315	0.356
	>3.0	26	17 (18.7)	9 (9.9)		
CA724 (U/ml)	≤5.3	5	1 (7.7)	4 (30.8)	0.506	1.000
	>5.3	8	3 (23.1)	5 (38.5)		

*HIPEC* hyperthermic intraperitoneal chemotherapy, *FIGO* International Federation of Gynecology and Obstetrics

**Table 3:** Correlation between CEP55 expression and clinicopathological characteristics of patients with EOC

Variable	CEP55 expression	
	Association coefficient	*P* value
FIGO Stage	0.550	<0.001
Lymph node metastasis	0.328	<0.001
Intraperitoneal metastasis	0.414	<0.001
Tumor recurrence	0.607	<0.001
Vital status (at last follow-up)	0.557	<0.001
Differentiation grade	0.251	<0.001
Residual tumor size (cm)	0.235	<0.001
Neoadjuvant chemotherapy	0.239	<0.001
Cytoreductive surgery	0.158	0.020
Ascites see tumor cells (+)	0.157	0.020
CA153 (U/ml)	0.289	<0.001

*FIGO* International Federation of Gynecology and Obstetrics, *EOC* epithelial ovarian carcinoma

### Survival analysis

To identify factors with potential prognostic significance in ovarian patients, univariate analysis for each variable was performed in relation to the survival time. In our univariate analysis, stepwise inclusion of variables in the model indicated that significant prognostic factors included CEP55 protein level, lymph node metastasis, intraperitoneal metastasis, FIGO stage, differentiation grade, recurrence, age, CA153 serum level, and neoadjuvant chemotherapy. In addition, multivariate Cox regression analysis revealed that CEP55 protein level, lymph node metastasis, intraperitoneal metastasis, FIGO stage, recurrence, age, and neoadjuvant chemotherapy were indeed independent prognostic markers for ovarian cancer (Table 4).

**Table 4:** Univariate and multivariate analyses of prognostic factors in EOC using a Cox-regression model

	Univariate analysis			Multivariate analysis		
	No. patients	*P*	Regression coefficient (SE)	*P*	Relative risk	95 % confidence interval
Lymph node metastasis						
Absent	173	<0.001	4.297 (0.215)	<0.001	2.262	1.429–3.581
Present	40					
FIGO stage						
I	59	<0.001	3.871 (0.135)	<0.001	3.151	2.179–4.558
II	36					
III	100					
IV	18					
CEP55						
Low expression	114	<0.001	11.729 (0.264)	0.015	2.705	1.209–6.054
High expression	99					
Recurrence						
No	121	<0.001	9.132 (0.242)	0.003	4.419	1.659–11.768
Yes	92					
Age (years)						
<53	111	0.036	1.520 (0.199)	<0.001	2.574	1.662–3.985
≥53	102					
Differentiation						
G1/G2	128	0.005	1.746 (0.199)	0.822	0.953	0.624–1.455
G3	85					
Intraperitoneal metastasis						
No	110	<0.001	4.415 (0.223)	0.003	2.023	1.262–3.243
Yes	103					
CA153 (U/ml)						
<38	82	<0.001	2.376 (0.231)	0.368	0.793	0.479–1.314
>38	121					
Neoadjuvant chemotherapy						
No	158	0.036	1.561 (0.212)	0.011	0.555	0.352–0.873
Yes	55					

*EOC* epithelial ovarian carcinoma, *FIGO* International Federation of Gynecology and Obstetrics

**Fig. 5:**
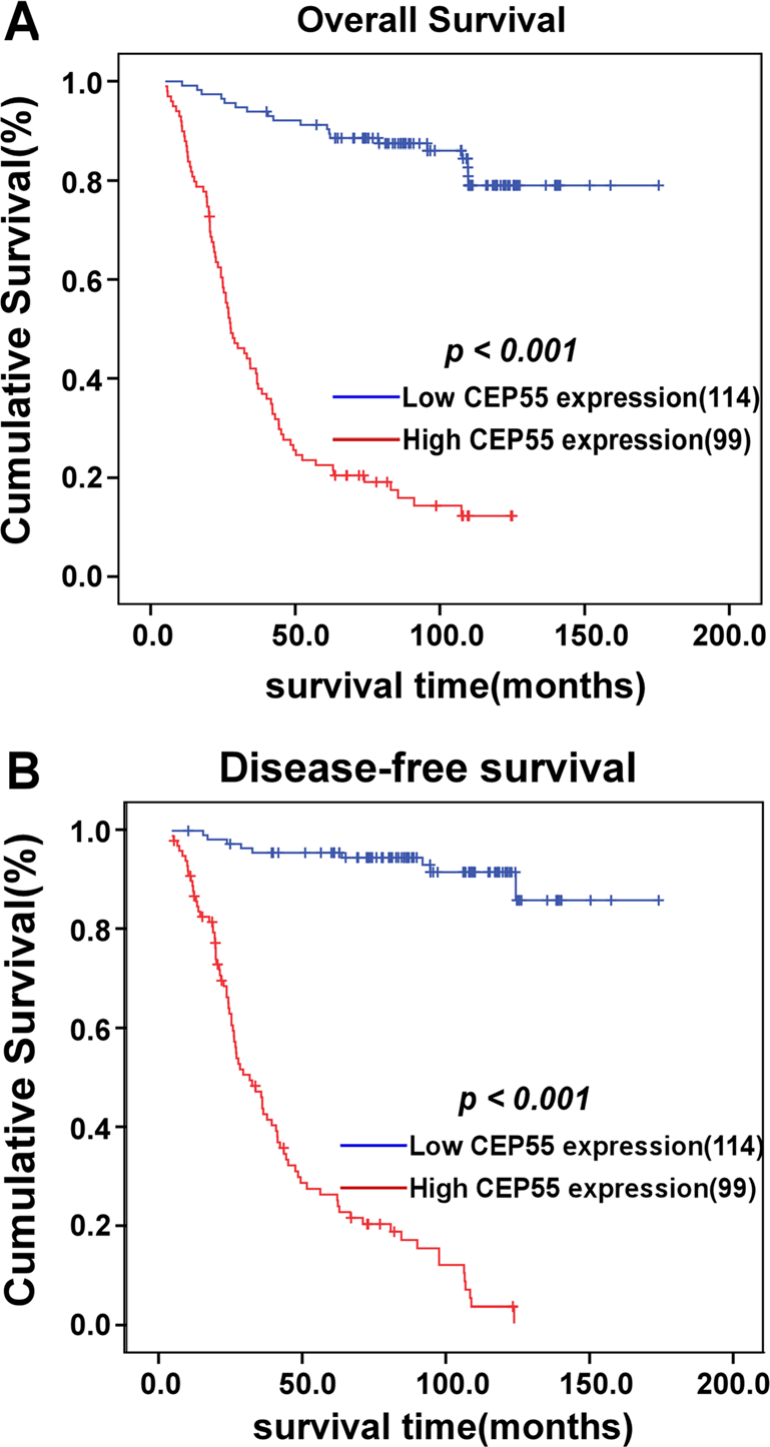
Survival curves of patients with EOC, subdivided according to CEP55 protein expression (log-rank test). **a**, **b** OS (**a**) and 5-year DFS (**b**) rates for cases with high CEP55 expression versus those for cases with low CEP55 expression levels in all patients

**Fig. 6:**
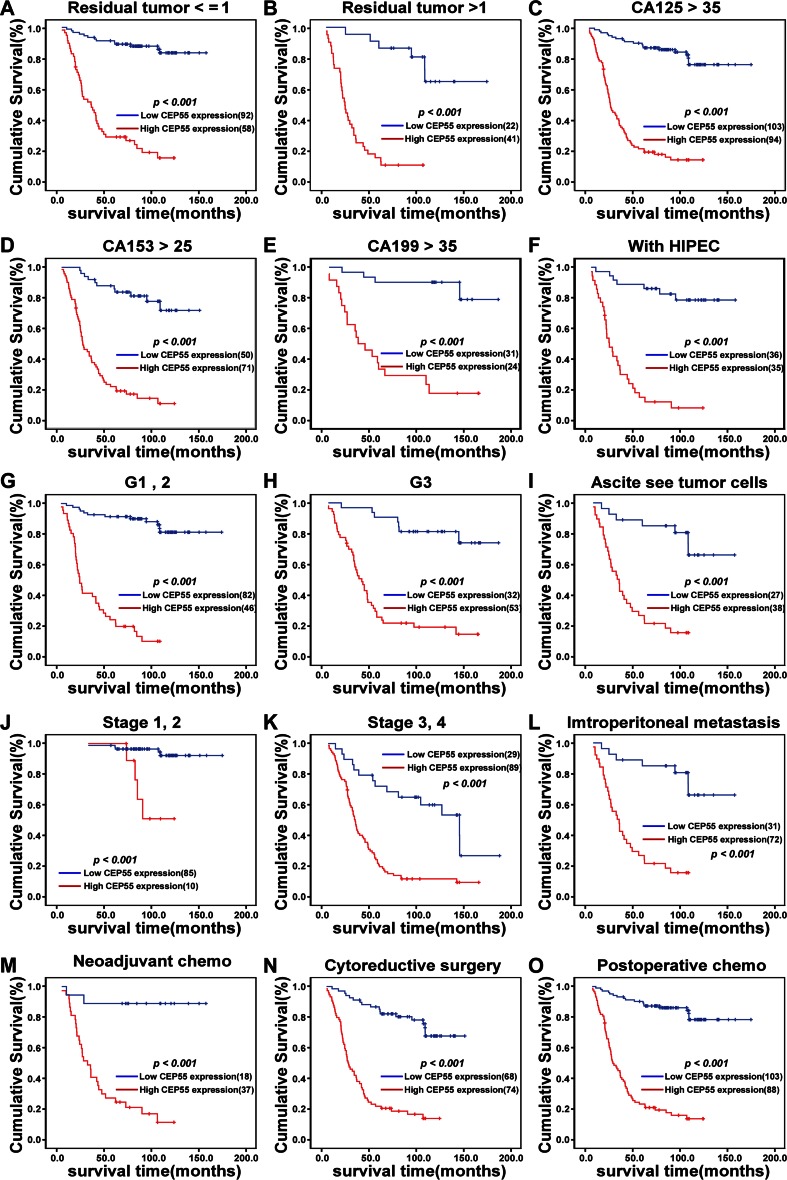
Kaplan*–*Meier curves of univariate analysis data (log-rank test) in select patient subgroups. Shown are OS rates for patients with high versus low CEP55 expression who had **a** residual tumor size ≤1 cm, **b** residual tumor size >1 cm, **c** CA125 >35 U/ml, **d** CA153 >25 U/ml, **e** CA199 > 35 U/ml, **f** received HIPEC, **g** differentiation grade 1–2, **h** differentiation grade 3, **i** ascites see tumor cells, **j** FIGO stages I-II, **k** FIGO stages III–IV, **l** intraperitoneal metastasis, **m** received neoadjuvant chemotherapy, **n** received cytoreductive surgery, and **o** received postoperative chemotherapy

To further determine the value of CEP55 expression in predicting survival of ovarian cancer patients, Kaplan–Meier analysis and the log-rank test were used in this study. The log-rank test showed that the survival time was significantly different between these two groups. As shown in Fig. 5, CEP55 expression in ovarian cancer patients was associated with survival time, with the patients expressing low CEP55 in their ovarian cancer lesions surviving much longer than those with high CEP55 expression (*P* < 0.001). The cumulative OS and disease-free survival (DFS) rates for the patients with high levels of CEP55 expression were 38.9 and 43.6 %, respectively, whereas these rates were 84.5 and 91.6 %, respectively, for patients with low or no CEP55 expression. Moreover, we analyzed the prognostic value of CEP55 expression in selected patient subgroups stratified according to characteristics of residual tumor size, serum CA125 level, serum CA153 level, serum CA199 level, differentiation, FIGO stage, intraperitoneal metastasis, and ascites see tumor size, as well as treatments of hyperthermic intraperitoneal chemotherapy (HIPEC), neoadjuvant chemotherapy, cytoreductive surgery, and postoperative chemotherapy. Patients with tumors exhibiting high CEP55 expression had a significantly shorter OS compared to those with low CEP55-expressing tumors in the residual tumor size ≤1 cm subgroup (log-rank test, *P* < 0.001, Fig. 6a), residual tumor size >1 cm subgroup (log-rank test, *P* < 0.001, Fig. 6b), CA125 >35 U/ml subgroup (log-rank test, *P* < 0.001, Fig. 6c), CA153 >25 U/ml subgroup (log-rank test, *P* < 0.001, Fig. 6d), CA199 >35 U/ml subgroup (log-rank test, *P* < 0.001, Fig. 6e), in those receiving HIPEC (log-rank test, *P* < 0.001, Fig. 6f), in the differentiation grade 1 and 2 subgroup (log-rank test, *P* < 0.001, Fig. 6g), in the differentiation grade 3 subgroup (log-rank test, *P* < 0.001, Fig. 6h), in those with ascites see tumor cells (log-rank test, *P* < 0.001, Fig. 6i), in the stage 1 and 2 subgroups (log-rank test, *P* < 0.001, Fig. 6j), in the stage 3 and 4 subgroups (log-rank test, *P* < 0.001, Fig. 6k), in those with intraperitoneal metastasis (log-rank test, *P* < 0.001, Fig. 6l), in those receiving neoadjuvant chemotherapy (log-rank test, *P* < 0.001, Fig. 6m), in those receiving cytoreductive surgery (log-rank test, *P* < 0.001, Fig. 6n), and in those receiving postoperative chemotherapy (log-rank test, *P* < 0.001, Fig. 6o).

### Specific silencing of CEP55 in SKOV3 and TOV-21G cells with CEP55 siRNA

**Fig. 7:**
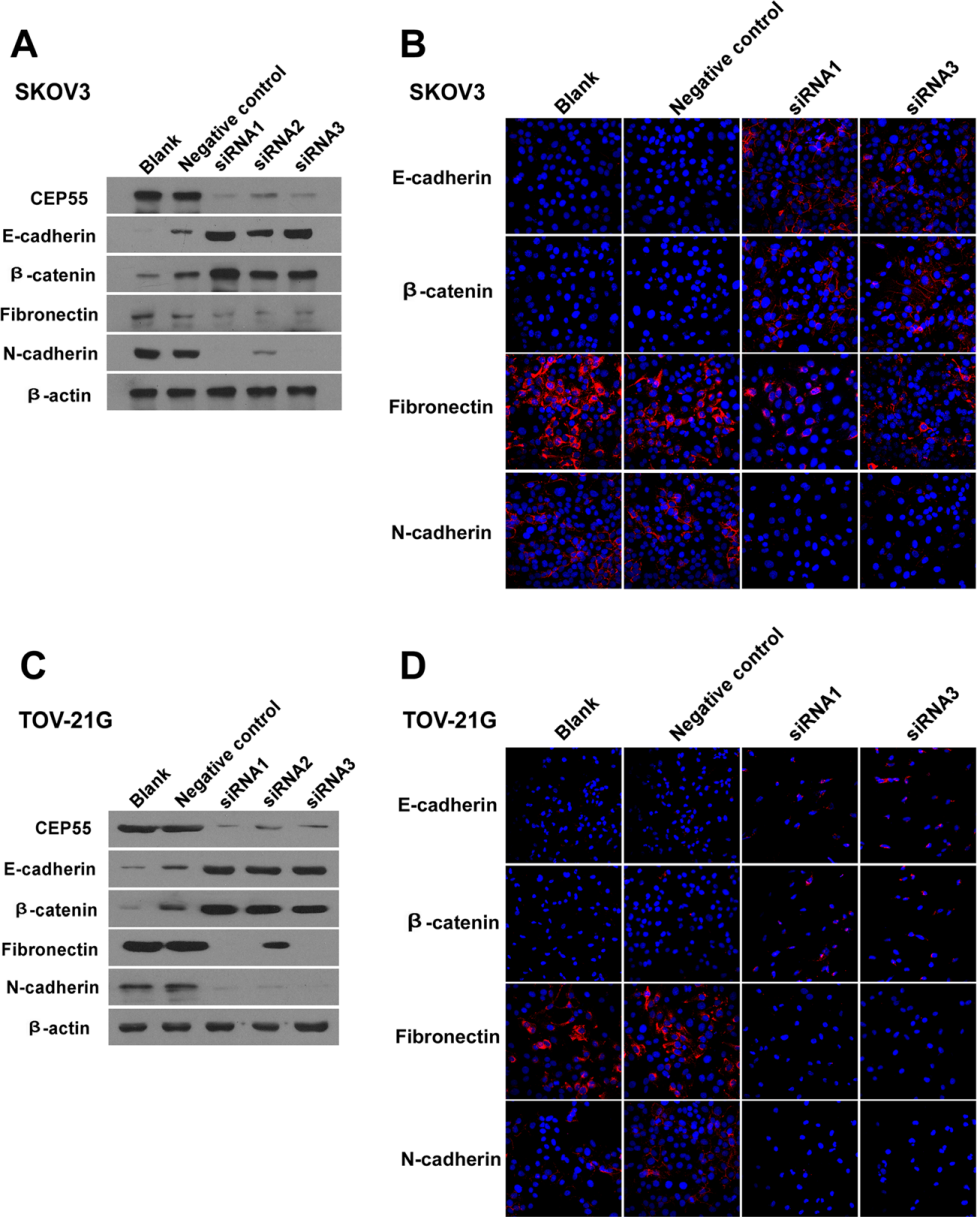
Downregulation of CEP55 reversed EMT. **a**, **c** Expression levels of CEP55 and EMT markers were analyzed by Western blotting. β-actin was used as the loading control. **b**, **d** Immunofluorescence assay of EMT markers (×200)

To further investigate the role of CEP55 in the invasion of ovarian cancer, we transfected SKOV3 and TOV-21G cells with 200 pmol siRNA for 24 h, which offered the best silencing efficiency in our preliminary experiments. Three different siRNA duplexes targeting CEP55 and a negative control siRNA were separately transferred into SKOV3 and TOV-21G cells. At 24 h after transfection, we examined the resulting phenotype using Western blot analysis. As shown in Fig. 7, CEP55 siRNA1 and CEP55 siRNA3 duplexes obviously reduced CEP55 protein in both ovarian cancer cell lines, and these more efficient siRNAs were therefore chosen for subsequent studies. The data were obtained from densitometric analyses of the ratio of CEP55 to β-actin protein levels (*n* = 5 independent experiments).

### Silencing of CEP55 inhibits migration and invasion in SKOV3 and TOV-21G cells

Cancer cell migration and invasion are directly related to metastasis, and CEP55 has been reported to be related to the processes of cell mobility and cancer metastasis [20]. In order to determine whether CEP55 induces epithelial–mesenchymal transition (EMT), we probed the cancer cell lines with epithelial and mesenchymal markers. As shown in Fig. 7, SKOV3 and TOV21G cells showed high CEP55 levels and the typical EMT phenotype, including downregulation of epithelial markers E-cadherin and β-catenin and upregulation of mesenchymal markers fibronectin and N-cadherin. As shown in Fig. 7a, c, silencing endogenous CEP55 in SKOV3 and TOV21G cells led to enhanced expression of epithelial markers and concomitant decreased expression of mesenchymal markers. The EMT phenotype was confirmed by immunofluorescent staining in SKOV3 and TOV-21G cells (Fig. 7b, d).

**Fig. 8:**
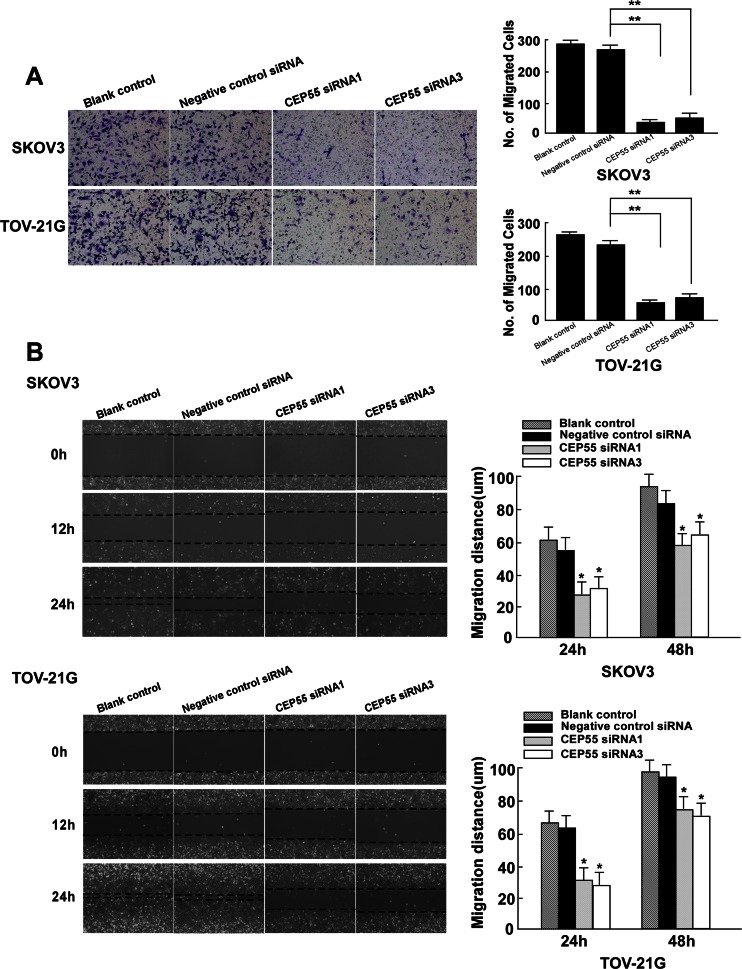
Downregulation of CEP55 repressed cellular motility and invasion. **a** Invasive properties induced by FBS were analyzed by the Boyden chamber invasion assay (×200, *P < 0.05). **b** Mobility of cells was measured by testing the rate of wound closure at 0, 12, and 24 h (×200) (*P < 0.05)

To further determine whether repression of CEP55 expression would inhibit SKOV3 and TOV-21G cell migration and invasion, a wound-healing assay and a cell invasion assay were performed on these two cell lines either untransfected or transfected with CEP55 siRNA1, CEP55 siRNA3, or negative control siRNA for 24 h. Results of the wound-healing assay revealed that the invasiveness of SKOV3 and TOV21G cells was dramatically hampered by the ablation of CEP55 (Fig. 8b). As shown in Fig. 8a, the number of SKOV3 cells that passed through the filter in the CEP55 siRNA1- and siRNA3-treated groups was remarkably lower than that in the untreated or negative control treated siRNA groups, indicating that inhibition of CEP55 expression suppressed SKOV3 and TOV-21G cell invasion in vitro.

## Discussion

To our knowledge, this study is the first to show that highly expressed CEP55 in patients with epithelial ovarian carcinoma was significantly correlated with clinical stage, lymph node metastasis, intraperitoneal metastasis, tumor recurrence, differentiation grade, residual tumor size, ascites see tumor cells, and serum CA153 level. Moreover, patients with aberrant CEP55 protein expression showed a tendency to receive neoadjuvant chemotherapy and cytoreductive surgery. The loss of CEP55 function mediated by RNAi in ovarian cancer cell models indicated that suppression of CEP55 repressed cellular invasion, demonstrating the involvement of CEP55 in enhancing the migratory and invasive activity in ovarian cancer cells. Furthermore, elevated expression levels of CEP55 protein served as independent prognostic factors for short OS and DFS rate in patients with EOC. In light of these findings, our results suggest that overexpression of CEP55 protein is associated with tumor aggressiveness and may represent an independent prognostic factor for clinical outcomes in EOC patients.

Recent studies have indicated overexpression of CEP55 as an important event in patients with certain types of cancer. Its elevated expression has been shown to be associated with carcinogenesis in bladder cancer, breast cancer, gastric cancer, and colon cancer [21–26]. Moreover, high CEP55 expression was found to promote progrowth signaling pathways resulting in cancer cell metastasis and poor patient prognosis [14, 23]. Altogether, these findings suggest that CEP55 may play an oncogenic role in cancer development and progression. In our cohort, we examined the expression of CEP55 mRNA and protein in ovarian cancer cell lines and ovarian cancer samples. We found that both CEP55 mRNA and protein showed aberrant expression levels in ovarian cancer tissues compared with noncancerous tissues, indicating that the overexpression of CEP55 occurred not only at the posttranscriptional level but also at the transcriptional level. Furthermore, we analyzed the correlation between CEP55 expression and the clinicopathological features of ovarian cancer patients. We found that CEP55 protein expression was significantly correlated with FIGO stage, tumor recurrence, differentiation grade, residual tumor size, ascites see tumor cells, serum CA153 level, lymph node metastasis, and intraperitoneal metastasis strongly supporting the hypothesis that this protein plays a role in the progression of ovarian cancer. Patients with CEP55 protein overexpression showed a tendency to accept neoadjuvant chemotherapy and cytoreductive surgery. In addition, univariate and multivariate analyses showed high CEP55 protein expression as a predictor of poor prognosis in these patients. Patients with elevated CEP55 expression showed a 38.9 % cumulative OS rate, which was significantly lower than that in patients with low expression levels of this protein (91.6 %). Taken together, these findings provide with essential, reliable evidence for the clinical significance of CEP55 as an independent prognostic marker to identify ovarian cancer patients with poor prognosis. Using CEP55 biomarker to identify patients with a higher risk of developing worse clinical outcome may thus help patients choose better treatment and reduce mortality.

To date, lymph node metastasis and intraperitoneal metastasis play important roles in determining the ovarian carcinoma staging. According to FIGO (2013), stage III is divided into three substages: stage IIIA, stage IIIB, and stage IIIC. Moreover, stage IIIA is divided further into substages IIIA1 and IIIA2. Ovarian cancer patients only with a positive retroperitoneal lymph node are classified as stage IIIA1, while patients with microscopic intraperitoneal metastasis are defined as stage IIIA2. Furthermore, patients who have macroscopic intraperitoneal metastasis are determined as stage IIIB and IIIC. In addition, recent studies reported that lymph node metastasis represents poor clinical prognosis for ovarian cancer patients [27]. Early diagnosis of intraperitoneal metastasis is pivotal for the survival of patients with EOC [28]. Therefore, having the ability to predict lymph node metastasis and intraperitoneal metastasis is critically important, but no ideal, preoperative marker has been established. In our study, we found that aberrant CEP55 protein expression was significantly associated with lymph node metastasis and intraperitoneal metastasis. Moreover, we observed a significant correlation between shorter OS and high CEP55 protein expression in the subgroup with intraperitoneal metastasis, which indicates that CEP55 may be a useful prognostic marker for such ovarian cancer patients. EMT has been found to contribute to invasion, metastatic dissemination, and acquisition of therapeutic resistance of cancer cells [29]. Several reports have shown that CEP55 plays an important role in regulating EMT. For example, increased CEP55 expression was reported to promote EMT in nasopharyngeal carcinoma via the osteopontin/CD44 pathway [23, 24]. Chen et al. [17] showed that CEP55 could regulate EMT through the CEP55/FOXM1/MMP-2 pathway in oral cavity squamous cell carcinoma. Moreover, overexpression of CEP55 was shown to regulate EMT through the VEGF-A/PI3K/AKT pathway in lung cancer [18]. Here, we suppressed CEP55 expression by siRNA and demonstrated, for the first time, that downregulation of CEP55 remarkably repressed ovarian cancer cellular invasion and reversed EMT. However, further studies are required to obtain a detailed picture of CEP55-related signaling pathways in regulating ovarian cancer EMT.

Although primary cytoreductive surgery followed by chemotherapy has been the standard treatment for advanced ovarian cancer for many years, neoadjuvant chemotherapy followed by interval debulking surgery has emerged as a new alternative treatment. National Comprehensive Cancer Network (NCCN) guidelines indicate that patients with a clear diagnosis of histopathology, patients with ascites cytology showing malignant tumor cells, as well as those with a physical examination and imaging examination or exploratory laparotomy determining that the tumor would be difficult to remove should accept neoadjuvant chemotherapy [30]. Recent studies have shown the benefit of neoadjuvant chemotherapy for advanced ovarian cancer patients. Kang et al. [31] found that neoadjuvant chemotherapy was not only equally effective but also a substantially safer strategy compared with standard treatment. Neoadjuvant chemotherapy, followed by surgical cytoreduction, is reportedly a promising treatment strategy for the management of advanced EOC [32]. Interestingly, ovarian cancer patients in our study with aberrant CEP55 protein expression had a tendency to accept neoadjuvant chemotherapy. Moreover, upregulation of CEP55 protein expression was significantly associated with ascites see tumor cells, which is one of the indications for accepting neoadjuvant chemotherapy. In a more detailed analysis of survival, we observed a significant correlation between shorter OS and high CEP55 expression in the subgroup with neoadjuvant chemotherapy. This finding suggests that CEP55 may be a useful prognostic marker for ovarian cancer patients with neoadjuvant chemotherapy.

Primary cytoreductive surgery and adjuvant chemotherapy are standard treatments for advanced ovarian cancer [33]. In our cohort, patients with high expression of CEP55 protein showed a significant tendency to obtain cytoreductive surgery. Furthermore, we found that higher CEP55 protein expression was correlated with a significantly shorter OS in the cytoreductive surgery subgroup. However, no correlation was observed in the subgroup without cytoreductive surgery. This result indicates that CEP55 protein expression is a more significant predictor of prognosis for ovarian cancer patients who require cytoreductive surgery. Currently, postoperative chemotherapy is the main treatment for ovarian cancer patients who have been treated with cytoreductive surgery. Our study identified a significant correlation between shorter OS and high CEP55 expression in the postoperative chemotherapy subgroup. This finding suggests that CEP55 may be a useful prognostic marker for ovarian cancer patients with postoperative chemotherapy. One of the most distinct features of EOC is the tendency to disseminate into the peritoneal cavity and remain confined to the peritoneum and intra-abdominal viscera. The peritoneal barrier enables the targeted delivery of chemotherapy directly to the peritoneal tumors [34], which makes it an ideal target for locoregional therapy. In randomized trials, intraperitoneal therapy following frontline surgery has shown a significant impact on survival [35]. Improved long-term results can be achieved in highly selected patients using cytoreductive surgery in combination with HIPEC [36]. For advanced ovarian cancer, a curative therapeutic approach combining optimal cytoreductive surgery and HIPEC should be considered as it may achieve long-term survival in patients with a poor prognosis, even in those with chemoresistant disease [37]. Here, our data showed that patients with high CEP55 protein expression did not have a significant tendency to receive HIPEC. However, a significant correlation was found between higher CEP55 expression and shorter OS in the HIPEC subgroup, which indicates that CEP55 expression is an important prognostic factor of ovarian cancer patients who accept HIPEC.

## Conclusions

In this cohort, we demonstrated the upregulated expression of CEP55 in EOC cells and surgical specimens and reported, for the first time, the correlation of CEP55 protein expression with clinical stage, lymph node metastasis, intraperitoneal metastasis, tumor recurrence, differentiation grade, residual tumor size, ascites see tumor cells, serum CA153 level, as well as prognosis in patients with EOC. Patients with aberrant CEP55 protein expression showed tendencies to receive neoadjuvant chemotherapy and cytoreductive surgery. Moreover, CEP55 may induce ovarian cancer lymph node metastasis through regulating EMT. Taken together, our results suggest that CEP55 may be a marker predicting unfavorable outcomes in ovarian carcinoma and plays a significant role in the migration and invasion of human EOC.
